# Molecular diagnosis and phylogenetic analysis of human papillomavirus type-16 from suspected patients in Pakistan

**DOI:** 10.1186/s13027-016-0047-z

**Published:** 2016-01-12

**Authors:** Abdullah Abdullah, Muhammad Qasim, Muhammad Shafiq, Muhammad Ijaz, Shamsa Parveen, Shahnaz Murtaza, Qamar Javed, Salman Akbar Malik, Shahida Husain Tarar, Sajid Mehmood, Abdul Sami, Syed Muhammad Saqlan Naqvi, Muhammad Zeeshan Hyder

**Affiliations:** Department of Biosciences, COMSATS Institute of Information Technology, 45550 Islamabad, Pakistan; Department of Biochemistry, Quaid-i-Azam University Islamabad, Islamabad, Pakistan; Department of Clinical Medicine and Surgery, University of Veterinary and Animal Sciences-Lahore, Lahore, Pakistan; Department of Obstetrics and Gynecology, Benazir Bhutto Hospital Rawalpindi, Rawalpindi, Pakistan; Department of Clinical Diagnostics, Nuclear Oncology and Radiotherapy Institute, Islamabad, Pakistan; Department of Obstetrics and Gynecology, Nawaz Sharif Medical College, University of Gujrat, Gujrat, Pakistan; Department of Biochemistry and Molecular Biology, Nawaz Sharif Medical College, University of Gujrat, Gujrat, Pakistan; Department of Biochemistry, PMAS Arid Agriculture University Rawalpindi, Rawalpindi, Pakistan; Department of Integrative Engineering, Chung Ang University, Seoul, South Korea; Immune Network Pioneer Research Centre, School of Medicine, Ajou University, Suwon, South Korea

**Keywords:** Cervical carcinoma, Sexually Transmitted Diseases, Papillomavirus, Phylogenetic, Molecular epidemiology

## Abstract

**Background:**

Human Papillomavirus (HPV) is well known pathogen that can cause benign and malignant tumors in humans, yet there is very little information regarding HPV types prevalent in Pakistan.

**Methods:**

A total of 92 cervical secretions were collected from suspected married female patients and used for DNA isolation using a novel isolation method. The samples were tested through Polymerase Chain Reaction (PCR) using already reported primers MY09/MY11, GP5/GP6, GP5+/GP6+, CP65/CP70, CP66/CP69 and SPF1/SPF2 and with those developed in this study including HRT1 and HRT2 primer sets for typing HPV types and HACTB primer set for human beta actin gene as internal positive control. Sequencing and phylogenetic analyses were performed for two isolates to determine circulating HPV types.

**Results:**

PCR with HRT1 and HRT2 indicated 2 (2.17 %) patients were positive for HPV type- 16 while 1 (1.08 %) with HPV type 18. Sequencing and phylogenetic analysis of isolates confirmed HPV type-16 in genus alpha 9 which have 99 % homology with already reported HPV from Japan and Costa Rica.

**Conclusion:**

This is the first report of HPV type-16 genus alpha 9 in Pakistan and the reported assay and sequence data will serve as valuable tools in further epidemiological studies for HPV surveillance to improve public health, especially of females in Pakistan.

## Background

Human papillomaviruses (HPVs) are diverse, non-enveloped, double stranded DNA viruses which are sexually transmitted and belong to family *Papillomaviridae*. The family *Papillomaviridae* is very large and contains 16 genera [1] with virus species which infects humans, non-human mammals and reptiles. More than 120 species of this family infect humans belonging to five genera including *Alphapapillomavirus*, *Betapapillomavirus*, *Gammapapillomavirus*, *Mupapillomaviurs* and *Nupapillomavirus* of which genus *Alphapapillomavirus* is the biggest. About fifteen HPV types including HPV-16, −31, −33, −35, −52, −58, (belonging to species *Human papillomavirus 16*), −18, −39, −45, −59, −68, (*Human papillomavirus 18*), −51, −82 (*Human papillomavirus 26*) −73 (*Human papillomavirus 34*) and −56 (*Human papillomavirus 53*) [1] are considered “high risk” due to their association with malignant transformation [2, 3]. The HPV type-16, followed by HPV type-18 are reported to be associated with 70 % of invasive cervical cancer worldwide [4].

Infection with human papillomaviruses is directly associated with ano-genital cancer in both males and females [5, 6]. The cervical cancer associated with HPV infection is the second most common cancer in women worldwide, with 8.2 % mortality rate [7], and is the major cancer of women in most of the developing countries [8] where majority of the cervical cancer cases occur [7] due to the lack of health facilities to diagnose and treat the disease. Pakistan is one of the developing countries where social and religious taboos, the stigma associated with sexually transmitted diseases (STDs), and economic barriers are major hurdles in the fight against STDs. Furthermore, the awareness of STIs among sex-workers especially, and among nursing staff and interns in tertiary-care hospitals, in general [9] is almost zilch. The social demographics warrant wide spread epidemic of STIs in general population of Pakistan [10, 11] as anticipated for Human immunodeficiency virus (HIV) [12, 13].

HPV infection has been implicated in carcinomas of cervix [14–16], oral cavity [17] and lungs [18] in Pakistan as well as its DNA has been detected in general population [15, 19]. The studies aiming to investigate prevalence of HPV utilize general primers for detection of HPV DNA. Currently most commonly used universal primers for HPV types are MY09/MY11 [20], GP5/GP6 [21], GP5+/GP6+ [22], CP65/CP70, CP66/CP69 [23], and SPF1/SPF2 [24] designed during 1990s or earlier. These primers were designed when sequence data of many of the currently known HPV genomes were not available as well as computation algorithm and power to reveal genome wide conserved regions were also not sufficiently advanced. Therefore, need existed to develop primers based on more rigorous analyses with larger sequence dataset which can not only detect more HPV types as well as can amplify sufficient proportion of HPV genome suitable for sequencing and phylogenetic analysis for rigorous confirmation of infecting type. Here we describe the development of a cheap method for HPV DNA isolation from small quantities of cervical secretions and novel primers designed for quality amplification of high risk HPV types. We also reported the sequence and phylogenetic analysis of two HPV isolates from Pakistan, which provided baseline information of molecular epidemiology of HPV in Pakistan.

## Results

The DNA isolation method developed in this study consistently isolated about 0.5 to 1.0 μg of DNA from cervical secretions with values greater than 1.7 for absorbance ratio of 260/280 nm. The PCR analyses indicated that of 92 suspected married female patients of diverse age group and ethnicity, only 2 (2.17 %) patients tested positive for HPV type-16, while 1 (1.08 %) was tested positive with HPV Type 18. The PCR amplification with already reported primer sets MY09/MY11, GP5/GP6, GP5+/GP6+, CP65/CP70, CP66/CP69, and SPF1/SPF2 were performed but showed no positive result, although the human actin beta gene was positively amplified using HACTB primer set. When these samples were tested with HRT1 and HRT2 novel primer sets, 3 out of 92 samples were found positive for HPV, two with HRT1 while other from HRT2 primer sets. Two out of these three positive samples were from a subset of two patients that were later diagnosed with cervical carcinoma, indicating a positivity of 100 % (2 out of 2) in patients with cervical carcinoma. The positive samples using HRT1 were further cloned and sequenced which is deposited in the GenBank through accession number JX073135 and KU212390 under the name of HPV Pak01 and HPV Pak02 isolates respectively, while sample positive using HRT2 was not sequenced due to paucity of the infected DNA.

**Fig. 1:**
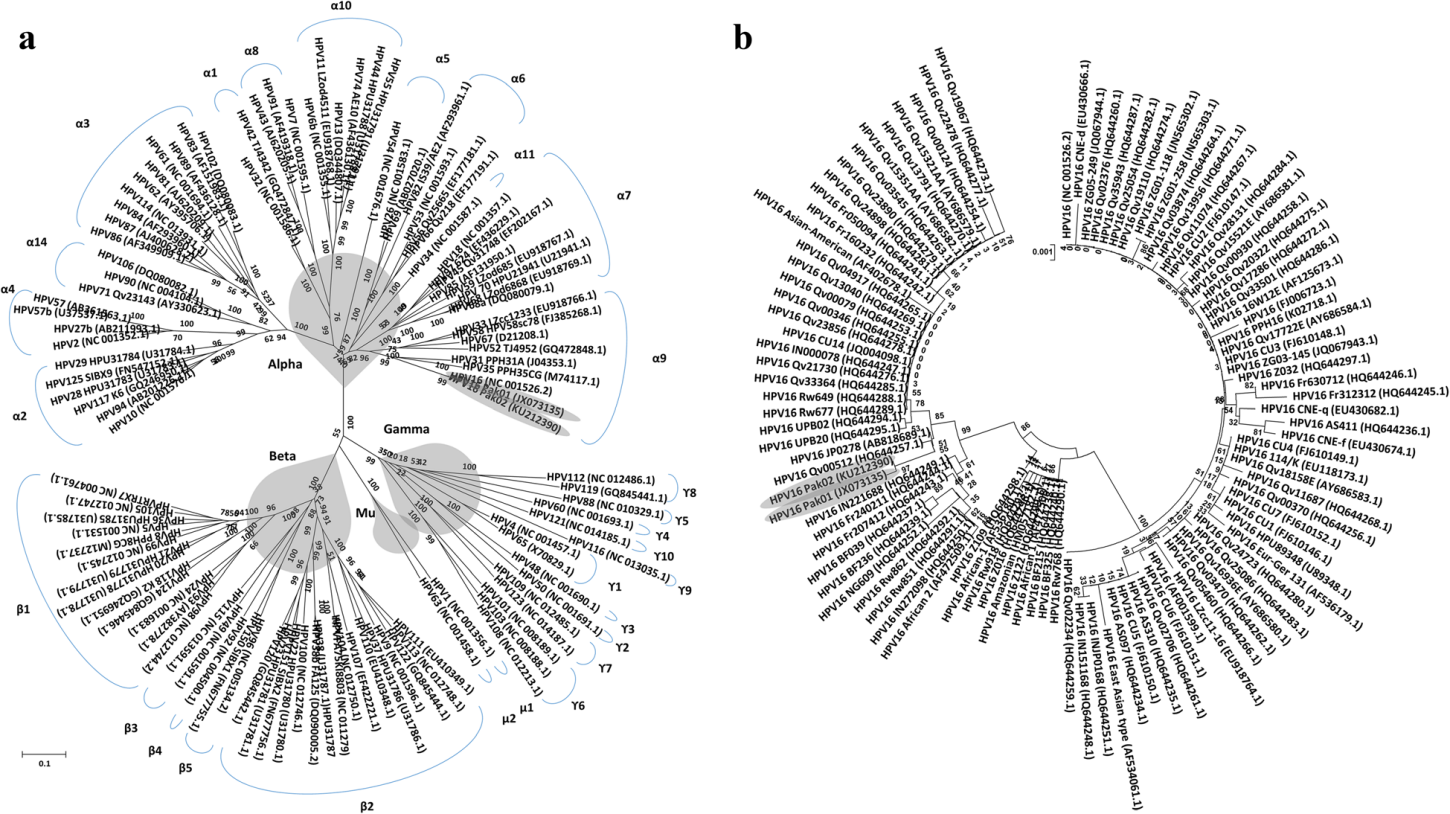
Phylogenetic analyses of HPV isolate Pak01. **a**. The radiating NJ tree of HPV Pak01 and Pak02 with other HPV types having full-length genome sequences available from GenBank was constructed using MEGA version 5.05 [57]. The HPV Pak01 and Pak02 isolates (highlighted in gray) cluster with HPV type 16 reference sequence (accession no. NC_001526.2) with 100 % bootstrap support, in HPV16 species which include HPV types 16, 31, 35, 33, 52, 58 and 67 [1] and collectively belongs to α9 in genus alpha [26]. The annotation of species and genera in the tree follows Bernard and colleagues [26]. The bar indicates substitutions per site. **b**. The circular NJ tree was generated by phylogenetic analysis of HPV Pak01 and Pak02 with HPV 16 isolates having full-length genome sequences available from GenBank. The HPV Pak01 and Pak02 isolate (highlighted in gray) clusters with HPV type 16 isolates JP02789 from Japan and Qv00512 from Costa Rica [29]. The bracket parenthesis indicates substitutions per site

The sequence analysis of 1461 bp fragment amplified by HRT1 primer set, of both Pak01 and Pak02, indicates that both are more than 99 % identical to each other with only one synonymous mutation at 426 nt from G in Pak01 to A in Pak02. The phylogenetic analysis with sequences of other HPV types, revealed that both the isolates clusters with 100 % bootstrap support with HPV type 16 reference sequence [25], among HPV 16 species (Fig. 1a) in genus alpha 9 (α9) [26]. Their relationship with other HPV type 16 isolates were further determined by BLAST analysis [27]. All the sequences of HPV 16 with full sequence coverage in BLAST analysis were extracted from GenBank and used for phylogenetic analysis (Fig. 1b). The phylogenetic analysis HPV 16 isolates indicated that HPV Pak01 and Pak02 clustered with 85 % bootstrap value and more than 99 % homology with isolates JP0278 and Qv00512 originated reportedly form Japan [28] and Costa Rica [29] respectively.

## Discussion

Pakistan with a population of more than 180 million according to Population Census Organization of Pakistan (http://www.pbs.gov.pk/content/population-census) is the sixth most populous country in the world. Breast cancer is one of the most common cancers in the country with 13.8 % incidence and 10.2 % mortality followed by cervical cancer with 8.4 % incidence and 7.2 % mortality [7]. In current study we detected HPV in 3.26 % of cases with gynecological problems, later on two of these three positive patients were diagnosed cervical cancer. Previously, the HPV positivity of 2.8 % in general population, 27.3 % in patient with cervical abnormalities [15] and 18 % [16] to 98.33 % [14, 30] in cervical carcinoma patient has been reported for Pakistani population. Only, the primers sets (HRT1 & HTR2) developed in this study showed positive results as compared to previously reported universal primers designed during 1990s.

HPV infection has been implicated in breast [1, 31] and cervical cancers [32, 33]. The detection of HPV types in cancer is commonly done by different PCR assays which utilize various universal primers including MY09/MY11 [20], GP5/GP6 [21], GP5+/GP6+ [22], CP65/CP70, CP66/CP69 [23], and SPF1/SPF2 [24] designed during 1990s or earlier. The specificities of these assays differ towards different types [34–36], which pose inconsistencies in detection and accurate determination of prevalence of various HPV types [34, 37, 38]. These inconsistencies might be one of the factors in the controversial reports of the presence of HPV in malignant tumors by some studies [5, 39–44] and absence by others^,^[45–48]. Therefore, development of primers capable of detecting more types is a crucial need. In addition, for epidemiological purposes it is of great importance to know the genetic sequence variability associated with virus population in that particular demographic and geographic region. These universal primers produce PCR products of less than 500 bp which is not sufficient for phylogenetic based confirmation in particular for HPV which have low mutation [49] but significant recombination rates [50].

The HRT1 and HRT2 primer sets developed in this study are based on more rigorous analyses with larger sequence data set compared to already developed primers. An in-silico analysis using BLAST searches in non-redundant database indicated that both these primer set together can theoretically amplify about nine different HPV types belonging to high risk category, while the other primer sets are only capable of theoretically detecting seven (MY09/MY11) and four (GP5+/GP6+) high risk HPV types (Table 1). Interestingly, GP5 primer was not even been matched to any HPV sequence present in non-redundant database. These and other primers presented in Table 1 produce a PCR product of less than 500 bp except HRT1 and HRT2 primer sets which target a region of about 1460 bp and about 1200 bp respectively and provide sufficient sequence information to perform phylogenetic analysis for elucidation of HPV type reliably. Although thorough analysis with larger data set is required for an empirical comparison, nevertheless theoretical comparison using BLAST searchers and positive PCR amplification of two samples which were shown negative with previously reported primers, indicates the usefulness of these primer in HPV detection, specifically in those geographical regions where lack of information about genetic structure of circulating HPV types exists, such as Pakistan.

**Table 1:** In-silico analysis of primers sets using BLAST for detection of high risk human papillomavirus types

Primer sets	Sequence 5-′3′	Degenerate bases	Tm (°C)	Product size (Kb)	Reference	HPV Types										
						16	18	31	33	35	39	45	52	56	58	59
HRT1 F	GACAGCGGRTATGGCAATWSTGAAG	3	56	~1460	In this study	**+**	**−**	**+**	**+**	**+**	**−**	**−**	**+**	**−**	**+**	**−**
HRT1 R	CCACGTCCTTGAGAAAAAGGATTTCC	0														
HRT2 F	GAGACACACCWGARTGGATACA	2	56	~1200	In this study	**−**	**+**	**−**	**−**	**−**	**−**	**+**	**−**	**+**	**−**	**−**
HRT1 R	CCATAGTTCCTCGCATGTRTCT	1														
MY09	GCMCAGGGWCATAAYAATGG	3	45	~450	[20]	**+**	**+**	**+**	**+**	**−**	**+**	**−**	**−**	**−**	**+**	**+**
MY11	CGTCCMARRGGAWACTGATC	4														
GP5	TTTGTTACTGGTAGATAC	0	40	~140	[21]	**−**	**−**	**−**	**−**	**−**	**−**	**−**	**−**	**−**	**−**	**−**
GP6	GAAAAATAAACTGTAAATCA	0														
GP5+	TTTGTTACTGTGGTAGATACTAC	0	40	~145	[22]	**+**	**+**	**+**	**−**	**−**	**−**	**+**	**−**	**−**	**−**	**−**
GP6+	GAAAAATAAACTGTAAATCATATTC	0														
CP65	CARGGTCAYAAYAATGGYAT	4	55	~465	[23]	x	x	x	x	x	x	x	x	x	x	x
CP70	AAYTTTCGTCCYARAGRAWATTGRTC	7														
CP66	AATCARMTGTTTRTTACWGT	4	55	~385	[23]	x	x	x	x	x	x	x	x	x	x	x
CP69	GWTAGATCWACATYCCARAA	4														
SPF1	GCICARGGICAYAAYAATGG	5	45	~65	[24]	x	x	x	x	x	x	x	x	x	x	x
SPF2	GTIGTATCIACWACAGTAACAAA	3														

The non-redundant/nucleotide collection (nr) database at National Center of Biotechnology Information (NCBI at http://www.ncbi.nlm.nih.gov/) was searched with the sequence of each primer by nucleotide Blast [27] using default values except maximum target sequences value was selected to 20,000. (+) means that both of the primers in a pair have secured blast hits for a particular HPV types mentioned above, while (−) means that one or both of the primers in a set have not been able to secure any blast hit for a particular HPV types. Due to the presence of many degenerate bases in the primer sequences, the Blast analysis was not been successful for primer sets CP65 & CP70, CP66 & CP69 and SPF1 & SPF2 and is represented by (x). The GP6 primer showed no hit specific to HPV. The product sizes are not exact and may differ slightly depending on the HPV types. The results in the table are based on Blast analysis only and may differ considerably compared to published results or when used empirically, therefore, please refer to the given references for exact details of HPV types amplified by each primer set

In population of Pakistan, although studies have been reported for the detection of HPV with *in situ* hybridization assay [51] and by PCR assay using universal primers [14–18, 30], sequence of any isolate and confirmation of circulating HPV types by sequencing and phylogenetic analysis was never reported. The phylogenetic analysis of current study confirmed that isolate Pak01 from a female patient suffering from cervical cancer and Pak02 from a female in Gujrat, belong to HPV type-16, and falls under *Human papillomavirus 16* species in genus alpha 9 of *Papillomaviridae*. The close phylogenetic relationship and 99 % nucleotide identity of these isolates with isolates from Japan and Costa Rica [29] indicate the probable role of expatriates in the spread of HPV across the world. The role of expatriates in spreading STIs such as HIV has been already been reported [52, 53] which had lead HIV to a verge of big epidemic in Pakistan [12].

The majority of population in Pakistan is living in poor socio-economic demography with little accessibility to quality medical care. In addition, social and religious taboos and stigma associated with sexually transmitted diseases offer great hindrance in studying and managing STIs. The studies on STIs with tracing of infection origin through phylogenetic analysis can help in identify the active transmitting group as well as risk groups and may lead pinpoint interventions for early diagnosis and blocking of disease transmission to healthy population.

## Methods

In this study 92 married female patients that were suspected for HPV of diverse age group and ethnicity, reporting various gynecological problems at three hospitals including Nuclear Medicine, Oncology and Radiotherapy Institute (NORI), Islamabad, Benazir Bhutto Shaheed (BBS) Hospital, Rawalpindi and Nawaz Sharif Medical College (NSMC), University of Gujrat, Gujrat, were included. The study was approved by the ethical committees at COMSATS Institute of Information Technology (CIIT), NORI, BBS and NSMC. Cervical secretion was collected after taking informed consents from the patients with the help of gynecologist using aseptic vaginal cotton swabs. DNA was extracted using a novel CTAB-Guanidine thiocyanate method. Briefly, 0.5–1.0 ml of 2 % CTAB buffer (100 mM Tris-HCl pH 8.0, 2 % (w/v) CTAB, 20 mM EDTA, 1.4 M NaCl, and 1 % (v/v) β-mercaptoethanol) was added in the swab container and homogenized using swab stick with cervical secretion for 2–3 min, for maximum sample recovery. About 0.5 ml of the mixture was transferred into eppendorf tubes and was added with 0.5 ml of 4 M guanidine thiocyanate solution and 0.7 ml of chloroform, mixed thoroughly and centrifuged at 16,000 g for 10 min. 0.1 volume of 3 M sodium acetate was added to the supernatant and mixed which then followed by the addition of isopropanol in equal quantity and centrifugation at 16,000 g for 5 min. After discarding the supernatant, the DNA pellet was washed twice with 70 % (v/v) ethanol and dissolved in 0.1× TAE Buffer containing RNase. The appropriateness of DNA isolation was confirmed by PCR amplification of a fragment of human actin beta gene from chromosome number 7 (accession number NG_007992.1) using primer set {HACTB F: 5′-CACAGTAGGTCTGAACAGACTC-3′ (position 6742-6763) and HACTB R: 5′-AGTGATCTCCTTCTGCATCCTG-3′ (position 7568–7547) as positive control. The samples were amplified with MY09/MY11 following procedure described by Manos and colleagues [20], with GP5/GP6 following Snijders and colleagues [21], with GP5+/GP6+ as described by de Roda Husman and colleagues [22], with CP65/CP70, and CP66/CP69 following Berkhout and colleagues [23], and with SPF1/SPF2 as reported by Kleter and colleagues [24].

Two novel primer sets HRT1 and HRT2 were developed by finding conserved regions in HPV genomes using about 90 full-length genomic sequences belonging to high risk HPV types 16, 18, 31, 33, 35, 45, 52, 56, 58, 59, 68 and 69 downloaded from GenBank and then aligned using MAFFT version 6.864 [54] which utilizes progressive and iterative refinement heuristic methods for large genomic sequences. Depending upon the genomic variability two sets of primers were designed i.e. HRT1 {HRT1 F: 5′-GACAGCGGRTATGGCAATWSTGAAG-3′(position 1254–1278 of type 16 reference genome accession NC_001526.2) and HRT1 R: 5′-CCACGTCCTTGAGAAAAAGGATTTCC-3′ (position 2714–2689 of type 16 reference genome accession NC_001526.2) which can detect types 16, 31, 33, 35, 52 and 58 and HRT2 {HRT2 F: 5′-GAGACACACCWGARTGGATACA-3′ (position 1935–1956 of type 18 reference genome accession NC_001357.1) and HRT2 R: 5′-CCATAGTTCCTCGCATGTRTCT-3′ (position 3134–3113 of type 18 reference genome accession NC_001357.1) which can detect types 18, 45, 56, 59, 68 and 69.

PCR reaction contained about 50 ng DNA template, Taq buffer (10 mM Tris-HCl, pH 8.8, 50 mM KCl and 0.08 % (v/v) Nonidet P40) 1.5 mM MgCl2, 200 μM of each dNTPs, 1.5 units Taq DNA Polymerase (recombinant) (Fermentas, UAB Lithuania), and 50 pM of each primer. The thermal profile for all primer sets included pre-PCR denaturation at 96 °C for 3 min followed by 35 cycles of denaturing at 96 °C for 20 s, annealing at 56 °C for 20 s and extension at 72 °C for 2.5 min, and a final extension at 72 °C for 20 min. The PCR products were analyzed using standard 1 % agarose gel electrophoresis.

The PCR product of HPV 16 isolate was cloned into pTZ57R (InsTA Cloning Kit, Fermentas UAB Lithuania), according to the manufacturer’s instructions, and transformed into *Escherichia coli* DH5α cells by electroporation. The cells were selected on ampicillin containing Luria-Bertani (LB) agar plates and screened for β-glucoronidase expression. Plasmid DNA was extracted using a minipreparation protocol according to Sambrook and Russell [55], and confirmed by digestion with restriction endonucleases. The plasmid DNA containing cloned fragment from three different independent clones, was sequenced using commercial sequencing facility of Macrogen (Korea) using M13 universal primers and gene specific primers. The sequence data was compiled using DNA Dragon Sequence Assembler version 1.5.1 (Sequentix-Digital DNA Processing, Germany).

Phylogenetic analysis was performed on alignments produced through MAFFT version 6.864 [54] to obtain Neighbor-Joining (NJ) trees using Kimura’s two-parameter model [56] implemented in in the Molecular Evolutionary Genetics Analysis Program (MEGA) version 5.05 [57]. Phylogeny reconstruction test was performed using 1000 bootstrap replicates.

## Conclusions

In conclusion, the study suggests the presence of HPV in 3.26 % of cervical sample secretions analyzed from Pakistani patients using two novel primer sets i.e. HRT1 and HRT2 developed by rigorous analysis of conserved regions in a large data set of HPV genomes. Isolates Pak01 and Pak02 originated from two female patients belonging to different geographical regions were sequenced for the first time using HRT1 primer set and their phylogenetic analysis suggests their belonging to genus alpha 9 of HPV type-16. The reported primers and sequence generated in this study will serve as foundation for further epidemiological studies for HPV surveillance in Pakistan.
